# Voluntary Kids’ Meal Beverage Standards: Are They Sufficient to Ensure Healthier Restaurant Practices and Consumer Choices?

**DOI:** 10.3390/ijerph17155275

**Published:** 2020-07-22

**Authors:** Phoebe Harpainter, Sridharshi C. Hewawitharana, Danielle L. Lee, Anna C. Martin, Wendi Gosliner, Lorrene D. Ritchie, Gail Woodward-Lopez

**Affiliations:** Nutrition Policy Institute, University of California, Division of Agriculture and Natural Resources, Berkeley, CA 94704, USA; pharpainter@ucanr.edu (P.H.); shewawitharana@ucanr.edu (S.C.H.); dnilee@ucanr.edu (D.L.L.); anpmartin@ucanr.edu (A.C.M.); wgosliner@ucanr.edu (W.G.); lritchie@ucanr.edu (L.D.R.)

**Keywords:** healthy default, beverage, policy implementation, childhood obesity prevention, quick-service restaurant, SNAP-Ed

## Abstract

Many quick-service restaurants (QSRs) instituted voluntary kids’ meal default beverage standards (standards) between 2013 to 2017. Little is known about impacts of standards on QSR drive-through practices and on customer choices. This study assessed differences in restaurant practices including kids’ meal beverages shown on menu boards, offered by cashiers, and selected by customers in QSRs with and without voluntary standards. Observations (*n* = 111) and customer surveys (*n* = 84) were conducted in 2018 at QSRs with standards (*n* = 70) and without (*n* = 41) in low-income California, U.S. neighborhoods. Kids’ meal beverages on menu boards (*n* = 149) and offered by cashiers (*n* = 185) at QSRs with and without standards were analyzed using multilevel logistic regression. Significantly more menu boards at QSRs with standards (*n* = 103) vs. without (*n* = 46) featured only milk, water or unsweetened juice (65.1% vs. 4.4%; *p* < 0.001). Most cashiers at QSRs with standards and QSRs without (53.1%, 62.5%) asked what drink the data collector wanted rather than first offering default beverages. A small sample of customer interviews found that customers at QSRs with standards most commonly ordered juice (37.0%); at QSRs without standards, soda (45.5%). Although menu boards showed healthier kids’ meal beverages at QSRs with standards than without, cashier behavior was inconsistent. Results suggest additional measures (legislation, implementation support, enforcement) may be needed to ensure optimal implementation.

## 1. Introduction

On an average day, a third of U.S. children consume food and/or beverages from quick-service restaurants (QSRs), accounting for roughly 12% of their energy consumption [1]. Children’s restaurant meal consumption is associated with a higher intake of sugar-sweetened beverages (SSBs) [2]. SSB consumption is a major contributor to obesity and diabetes [3]. Among California children aged 2–5, drinking soda or other SSBs is more prevalent among low-income than higher-income children [4]. QSRs are statistically significantly more prevalent in low-income areas and account for a high proportion of kids’ meal sales [5,6]. Instituting healthy default beverage (HDB) policies for kids’ meals (bundled meals for children with a beverage included, sold at a single price) at food retailers is considered one of the top five strategies to reduce SSB consumption and increase water access and consumption among young children [7].

Healthy defaults are an attractive strategy for encouraging healthy choices: they do not require conscious selection of healthier choices from an array of options. Rather, healthy defaults, when implemented as intended, make the healthy default choice either the only or the most effortless choice [8]. Effective implementation of a healthy default beverage strategy in the physical QSR setting would likely need to apply to all points at which beverages are marketed to customers, including options listed on menu boards, kiosk screens and verbally offered by cashiers during the ordering process [9]. By definition, the healthy default beverages would either be provided by default (i.e., without presenting other options) or would be the only options presented absent a request from the customer for other options [10].

With the implicit, and in two cases explicit, intent to “…help families and children make informed choices in keeping with balanced lifestyles” and to “increase customers’ access to … low-fat dairy, and water”, several QSR brands instituted voluntary HDB standards (herein referred to as “standards”) between 2013 and 2017 [11,12]. Instead of soda with kids’ meals, all committed to offer various combinations of milk and/or chocolate milk, juice and water as default options (below). In 2018, the American Beverage Association, a trade group representing the U.S. beverage industry, stated that “…parents are more than capable of making the food and beverage choices that are best for their families…we have repeatedly heard from parents that they believe that water, milk or juice are the best options” and indicated willingness to work with restaurants on adopting standards [13].

- McDonald’s (2013): water, milk or juice in Happy Meals [12];
- Subway (2014): low-fat or non-fat milk or water [14];
- Wendy’s (2015): 1% white or chocolate milk, bottled water and 100% juice [15];
- Burger King (2015): fat-free milk, 100% apple juice and low-fat chocolate milk [11];
- Dairy Queen (2015): milk and bottled water [16];
- McDonald’s (2017): began offering a low-calorie, 42% apple juice product (herein referred to as “diluted juice”) in addition to water and milk in Happy Meals [17].

Furthermore, the state of California, and several cities and counties in other parts of the U.S., have passed HDB legislation (herein referred to as “HDB law(s)”) for restaurants and many other jurisdictions are considering doing so [18,19]. The California law went into effect on January 1, 2019 and allows only water and/or unflavored milk (or an equivalent non-dairy beverage) as default beverage options. California’s law explicitly states the intent of the law is: “…to support parents’ efforts to feed their children nutritiously” [19].

Evaluation and effective implementation are key pieces of the policy process [20]. Evaluation of the voluntary standards can provide insight into the development and effective implementation of these policies. While some studies have been conducted to understand impacts of the above standards and other healthy default restaurant programs, little is known about effects on cashier practices or drive-through menu boards [12,21,22]. An evaluation of McDonald’s U.S. menu boards found all showed only water, milk (including chocolate milk) or juice following implementation of standards, but did not examine how cashiers offered beverages with kids’ meals [12]. An evaluation of Kids LiveWell, a program to increase the number of nutritious menu items available to children, showed no overall reduction in SSBs as a proportion of beverages offered on kids’ menus, and mixed or no results for improvement of beverage calories offered on kids’ menus [21]. Changes in cashier practices were not investigated. Another study sampled QSRs that implemented standards between 2013 and 2015 [22]. By 2016, most listed healthier drinks with kids’ meals. However, all chains still included fountain drinks on menu boards at some locations, indicating inconsistent implementation of these standards. Between locations of each chain, discrepancies in how cashiers offered beverages were observed. Importantly, this study did not capture any drive-through data. Including drive-throughs in restaurant observations is critical since drive-through orders account for 60–70% of QSR business [23].

In California, many local health departments (LHDs) are utilizing CalFresh Healthy Living (CFHL; California’s Supplemental Nutrition Assistance Program-Education, or SNAP-Ed program) resources to support restaurants in implementing this SSB reduction strategy. This study was designed in part to inform and evaluate these efforts. Therefore, to address gaps in the evidence and inform the effective development and implementation of healthier default beverage policies, this study examined inside and drive-through practices and customer beverage choices in 2018 in QSRs with and without standards in low-income California neighborhoods. Specifically, we examined the two ways in which beverages are “offered” to customers who place their orders in-person at QSRs: (1) by options as listed or pictured on menu boards and (2) by options presented verbally by cashiers during the ordering process. We hypothesized that QSRs with standards would “offer” healthier beverages more consistently than QSRs without standards. Furthermore, we were interested in whether the previously mentioned intent of the standards, i.e., encouraging customers’ healthier beverage choices, was achieved. We hypothesized that consumers at QSRs with standards would choose healthier beverages than customers at QSRs without standards.

## 2. Materials and Methods

Of the 60 California LHDs invited to participate in the study, 11 agreed. Each LHD identified census tracts of programmatic interest within their county that qualified for CFHL (at least 50% of residents have incomes <185% of the federal poverty level).

Within the identified census tracts, 205 QSRs offering kids’ meals that included beverages were identified from Dun and Bradstreet data [24]. Selected QSRs were separated into two categories: with or without standards, based on stated company pledges or standards previously described in the introduction, evaluation of restaurant websites and/or calls made to locations of the QSRs.

The 205 QSRs were grouped into geographic clusters within each county. Within each cluster, QSRs were stratified according to with/without standards, then ordered randomly within each stratum. Since there were many fewer QSRs without standards in the sampling frame, QSRs without standards were oversampled to ensure adequate representation of both QSR types in the final sample. Due to the short time frame between passage of California’s HDB legislation in September 2018 and the required implementation date of January 1, 2019, once IRB approval and all preparatory activities were completed, data collection was limited to a three-week period in December 2018 (excluding December 24–25). This timing ensured that we were measuring the situation under voluntary standards independent of the mandatory legislation. Clusters were ordered to minimize travel time between clusters, thereby maximizing the number of QSRs where data could be collected during these three weeks. Data collection was scheduled from 2 p.m. to 7 p.m. on weekdays and 11 a.m. to 7 p.m. on weekends (times of day when more kids’ meal sales were expected). Restaurants were excluded from the study if, upon visiting, the restaurant did not meet the inclusion criteria: no longer open for business, and/or not a QSR, and/or did not offer a kids’ meal that included a beverage.

A QSR observation tool and customer survey were adapted from existing tools, developed, field-tested and revised by a team of social, behavioral and public health researchers and registered dietitians [25,26,27]. If the QSR had a drive-through, data collectors first observed the drive-through menu board and placed a kids’ meal order in the drive-through, then entered the QSR to observe the inside menu board and order a second kids’ meal. Menu board observations documented kids’ meal components and what kids’ meal beverages were listed or pictured. Orders were placed using a standardized ordering script and all beverages ever offered by the cashier were documented. Data collectors first asked for a kids’ meal without mentioning beverages. Cashier offerings following this statement were categorized as one of the following:

- Only water and/or unflavored milk;
- Water and/or unflavored milk and other drinks;
- Drinks other than water and/or unflavored milk;
- Asks what drink you want (open-ended);
- Does not ask what drink you want, cashier chooses beverage automatically.

If cashiers offered beverage options or asked what drink the data collector wanted, data collectors inquired what the beverage options were. All beverages offered were categorized as sugar-sweetened drinks (soda, sports, energy or fruit-flavored drinks); diet drinks; flavored milk; any juice; milk; water; or other.

Following the observation, data collectors requested manager permission to interview customers (over 18 years of age who purchased a kids’ meal inside for a child 12 years or younger and spoke English or Spanish). Surveys were interviewer-administered, took approximately 5 min and consent was obtained. Participating customers were asked what beverage was purchased with the kids’ meal and for demographic information. All survey respondents gave their informed consent for inclusion before they participated in the study. The study was conducted in accordance with the Declaration of Helsinki, and the protocol was approved by the UC Davis Institutional Review Board Committee for the Protection of Human Subjects (#1344930-2). After completing the survey, respondents were offered a reusable shopping bag. Customer surveys were attempted at each QSR until 5 surveys were collected or for a maximum of 2.5 h. Surveys were administered only inside, not in the drive-through, due to logistical challenges. With the exception of a few chains, it was observed that few customers ordered inside, even during higher-volume meal times. Data collectors discontinued customer surveys at QSR chains where less than one survey had been collected at several QSR locations and at independent QSRs—as the time required to survey a sufficient number of customers became prohibitive.

### Statistical Analysis

Differences between beverages shown on menu boards and beverages offered by cashiers in QSRs with and without standards were assessed using multilevel logistic regression adjusting for clustering by chain and by QSR, when inside and drive-through observations were made at the same QSR. Missing values were excluded from analysis. Due to small sample sizes, statistical analyses to assess differences in customer beverages ordered were not conducted. Analyses were conducted in 2019 using Statistical Analysis Software (SAS) version 9.4., SAS Institute Inc., Cary, NC, U.S.

## 3. Results

Data collectors visited the QSRs in each county in the prescribed order, according to criteria described in the methods section. They continued down the list and visited as many restaurants as possible within their assigned time frames. They were able to visit 126 (61.5%) of the 205 QSRs on the lists. Fifteen QSRs (11.9%) of 126 visited were excluded because they were not a QSR and/or did not offer a kids’ meal that included a beverage. Permission was not needed or sought for conducting the observations. The final analytic sample for restaurant observations included 111 QSRs.

Sampled QSR standards included combinations of flavored and unflavored low-fat, fat-free or reduced-fat milk; 100% juice or diluted juice and/or bottled water as defaults with kids’ meals. One hundred QSRs (90.1%) belonged to national chains (Table 1), representing 14 different QSR brands (data not shown). Almost two-thirds of QSRs sampled had standards (63.1%). 

A total of 149 menu boards were observed across all QSRs. Menu boards at QSRs with standards were significantly more likely than menu boards at QSRs without standards to feature unflavored milk (92.0% vs. 43.2%; *p* = 0.005) or water (60.0% vs. 32.6%; *p* = 0.036); and statistically less likely to feature an unspecified “Drink”/“Kids’ drink” (1.9% vs. 41.3%; *p* = 0.037) (Table 2). SSBs were displayed on about one-third of menu boards at QSRs with standards (29.1%) and without standards (38.6%). A significantly larger proportion of menu boards at QSRs with standards displayed only milk, water or unsweetened juice vs. those without (65.1% vs. 4.4%; *p* < 0.001). Milk, water and juice are common beverages allowed in kids’ meals under QSR standards. No menu boards in QSRs without standards showed only unflavored milk or water; 15 in QSRs with standards (15.5%) did. Statistical significance could not be determined because the model failed to converge due to a lack of variation in the data. Unflavored milk and water are the only allowable HDBs for kids’ meals as specified by the California law that went into effect after these data were collected [19].

A total of 185 kids’ meal orders (drive-through and inside) were placed by data collectors; 113 (61.1%) were placed at QSRs with standards and 72 (38.9%) at QSRs without standards. The most common scenario at both QSRs with and without standards was that cashiers asked what beverage the data collector wanted rather than offering specific beverages (53.1% and 62.5%, respectively) (Table 3). A significantly higher proportion of cashiers offered water, unflavored milk and other beverages at QSRs with standards vs. without (15.0% vs. 1.4%; *p* = 0.024). No cashiers at QSRs without standards initially offered only unflavored milk or water; 5.3% of cashiers at QSRs with standards did (model unable to estimate adjusted *p*-value due to a lack of variation in data). No significant differences were observed for beverages offered by cashiers when asked about options, except diet drinks (QSRs with standards, 38.8%; QSRs without standards, 58.8%; *p* = 0.042) (Table 3).

Of the sample of 111 QSRs, customer surveys were obtained from 33: 26 with standards and 7 without. At five QSRs, managers declined to allow the customer survey. At 72 QSRs, no surveys were collected primarily because no survey-eligible customers purchased a kid’s meal inside during the allotted time frame, or because no customers agreed to participate. Four customers refused; nine were ineligible. A total of 84 customer surveys were conducted and analyzed, most (86.9%) at QSRs with standards (Table 1). All surveys at QSRs with standards and 6 of the 11 surveys at QSRs without standards were obtained at chain QSRs. The majority (78.1%) were collected at a single QSR chain (data not shown). Only one survey was collected at an independent QSR (data not shown). The majority of respondents at both QSRs with standards and without reported ordering the kids’ meal for Hispanic/Latino children (58.9% and 81.8%) and male children (52.1% and 54.6%). At QSRs with standards, most children resided in the same city or ZIP code as the QSR (71.2%); most children at QSRs without standards resided in a different city or ZIP code (63.6%). Most respondents across all QSRs reported being the parent, primary caregiver or legal guardian of the accompanying child (82.2% and 90.9%, respectively).

The sample of customer surveys at QSRs without standards (*n* = 11) was too small to draw any significant conclusions. Juice was the most common drink ordered at QSRs with standards (37.0%); no customers at QSRs without standards ordered juice (Table 4). The proportion of customers ordering regular soda at QSRs without standards (45.5%) was nearly twice that at QSRs with standards (26.0%); the proportion that ordered other pre-sweetened drinks (not including flavored milk) was somewhat larger at QSRs without (36.36%) than with standards (28.8%) (Table 4). Few surveyed customers at QSRs with standards (6.9%) and none at QSRs without standards ordered unflavored milk.

## 4. Discussion

In terms of beverages offered on menu boards and by cashiers, the biggest contrast between QSRs with and without standards was in beverages offered on menu boards. Significantly more QSRs with standards included unflavored milk or water on their menu boards compared with QSRs without. In QSRs with standards, the kids’ meal beverages offered on the menu board tended to be consistent with QSR standards. As specified by their policies, 65.1% of QSRs with standards’ menu boards included only milk, water or unsweetened juice.

Despite these positive menu board findings, there remained much room for improvement. Many QSRs with standards still displayed beverages (like SSBs) inconsistent with their own standards. Other studies have found similar inconsistencies. Harris et al. reported 30–42% of inside menu boards listed fountain drinks with kids’ meals 1–3 years after implementation of voluntary standards prohibiting them [22]. These inconsistencies in the implementation of standards may be impacted by franchise vs. corporate ownership. Franchise owners may have the latitude to implement standards differently at their restaurants. For example, some franchise-owned Dairy Queen outlets do not receive corporate point-of-sale materials (e.g., menu boards) [16]. However, the franchise-owned vs. corporate-owned portion of our sample and of Harris et al.’s sample is unknown.

Even more striking was the infrequency of cashiers offering healthy beverages and how much room for improvement exists in cashier practices. Healthier beverages were more frequently offered initially by cashiers at QSRs with standards than without. Although our ordering data did not allow for comparison of cashier offerings to the exact combination of beverages allowed by voluntary standards, many more cashiers at QSRs with standards initially offered water, unflavored milk and/or other beverages than cashiers at QSRs without standards. However, the percentage of cashiers at QSRs with standards initially offering these beverages was much lower than the fraction of menu boards displaying these drinks, suggesting standards were much less effective at influencing cashier behavior than at influencing menu board content. Rather than offering specific beverages, most cashiers at both QSRs with and without standards asked “What drink would you like with that?”. In this interaction, the burden is on the customer to request a healthy drink. After data collectors probed for options, cashiers frequently mentioned beverages consistent and inconsistent with standards, with no significant differences between QSRs with standards and QSRs without standards except for diet drinks. These findings, though not directly comparable due to differences in methodology, are consistent with Harris et al., who observed wide variation in how cashiers offered beverages, compared with menu board changes, following the adoption of standards [22]. Although Harris et al. indicated they encountered open-ended questions from cashiers, frequency was not provided.

Although our customer survey sample was too small to support statistical analysis or draw conclusions, the observed differences in customers’ beverage orders at QSRs with standards and at those without suggest this may be an important area for future research. For example, in our small sample, very few customers ordered unflavored milk or water at any QSRs; yet at QSRs with standards, many more customers ordered unsweetened juice and many fewer ordered SSBs compared with customers at QSRs without standards. We include these limited data because relatively little customer kids’ meal beverage purchase data exist in the literature. Our results suggest that assessing whether standards, even when primarily limited to menu board changes, positively affect customer purchasing behavior warrants further attention. These results are consistent with industry analysis following voluntary changes McDonald’s made to U.S. menu boards, which found the percentage of customers that selected soda with kids’ meals decreased from 56% to 48% [28]. One study found nearly two-thirds of parents surveyed in 2010, 2013 and 2016 reported receiving a healthier beverage (low-fat unflavored milk, water, 100% juice) with a purchased kids’ meal at chain restaurants with standards, and that this proportion did not change significantly over time [6]. Another study in 94 theme park QSRs that implemented HDB options found that 67.8% of customers purchased HDBs with kids’ meals over a three-year period [29]. No customer purchase data have been reported following implementation of previous HDB laws. Future studies using QSR sales data would be instrumental in understanding the impact of HDB laws and standards.

### Limitations

The study sample, although large, stratified and randomly ordered within each stratum, was drawn from a convenience sample of low-income communities in 11 California counties, selected because their health departments were interested in participating in an intervention. Therefore, the results may not be generalizable to all QSRs. Furthermore, we did not have time to include all geographic clusters (which were not ordered randomly) within each county, possibly introducing bias. Although we collected menu board and ordering data in the drive-through, due to logistical challenges, we were not able to survey drive-through customers, thereby limiting the generalizability of the customer survey findings. Although we had sufficient sample sizes for the menu board and ordering data analyses, the number of customer surveys in QSRs without standards was insufficient to support statistical analysis. Customer survey results are suggestive but not conclusive. Finally, the observational design precludes conclusions regarding causality. Although the large and significant differences in practices observed in sampled QSRs after adjusting for covariates are compelling, these differences cannot be definitively attributed to the standards. 

Despite these limitations, our findings provide valuable information about both the potential influence of standards and weaknesses in the ways these standards are applied. These findings can inform future HDB laws and implementation of current HDB laws in California and other states to increase their effectiveness in improving customer beverage choices. 

## 5. Conclusions

QSRs with standards consistently offered healthier drinks with kids’ meals than QSRs without. Customers at QSRs with standards reported purchasing healthier drinks and less soda compared with customers at QSRs without standards. These results suggest standards were effective at positively influencing restaurant practices and customer behavior. However, not all QSRs followed their standards: much room for improvement remained. Additional intervention may be necessary to support full implementation of the standards and to maximize the impact on customer behavior. Jurisdictions passing HDB laws may need to provide education and outreach alongside enforcement to ensure full implementation.

Careful crafting of new HDB laws may facilitate effective implementation that expands on existing standards. We observed standards that were implemented primarily via menu boards but to a much lesser extent in cashier practices. Therefore, it may be important to make clear in legislative language how HDB laws apply to the way cashiers offer beverages. Although customers in our study at QSRs with standards made healthier choices than those at QSRs without standards, many ordered SSBs and most ordered juice, which is not allowed with California’s new HDB law [18]. Education for QSRs and effective enforcement is recommended. Education and promotion for customers may also be needed to encourage parents and children to choose HDBs.

## Figures and Tables

**Table 1:** Characteristics of sampled quick-service restaurants (QSRs), customer survey respondents and accompanying children ^a^.

Characteristics of QSRs (*N* = 111)	QSRs with Standards ^b^ (*n* = 70)	QSRs with No Standards ^b^ (*n* = 41)
Number of menu boards observed at QSR (*n*%)		
0 ^c^	3 (4.29)	12 (29.27)
1 (drive-through or inside)	31 (44.29)	12 (29.27)
2 (drive-through and inside)	36 (51.43)	17 (41.46)
Number of orders placed at QSR (*n*%)		
1 (drive-through or inside)	26 (37.14)	9 (21.95)
2 (drive-through and inside)	44 (62.86)	32 (78.05)
QSR type (*n*%)		
Chain	70 (100.00)	30 (73.17)
Independent	0 (0.00)	11 (26.83)
**Characteristics of Restaurants Where Customers were Surveyed (*N* = 33)**		
	**QSRs with Standards ^b^ (*n* = 26)**	**QSRs without Standards ^b^ (*n* = 7)**
QSR type (*n*%)		
Chain	26 (100.00)	6 (85.71)
Independent	0 (0.00)	1 (14.29)
**Characteristics of Respondents to Customer Surveys (*N* = 84)**		
	**Respondents at QSRs with Standards ^b^ (*n* = 73)**	**Respondents at QSRs without Standards ^b^ (*n* = 11)**
**Accompanying Child Demographics**		
Age, years (Mean (SE))	5.77 (0.34)	7.82 (0.49)
Race/ethnicity (*n*%)		
Hispanic/Latino	43 (58.90)	9 (81.82)
White	9 (12.33)	2 (18.18)
Mixed/Multiethnic	11 (15.07)	0 (0.00)
Asian/Pacific Islander	6 (8.22)	0 (0.00)
African-American/Black	2 (2.74)	0 (0.00)
Native American/Alaska Native	1 (1.37)	0 (0.00)
Other, not specified	1 (1.37)	0 (0.00)
Gender (*n*%)		
Male	38 (52.05)	6 (54.55)
Female	34 (46.58)	5 (45.45)
Other/Non-binary	1 (1.37)	0 (0.00)
Residence (*n*%)		
Located in the same city or ZIP code that contains city of restaurant	52 (71.23)	4 (36.36)
Not located in the same city or ZIP code that contains city of restaurant	19 (26.03)	7 (63.64)
Missing	2 (2.74)	0 (0.00)
**Survey Respondent Demographics ^d^**		
Highest level of education (*n*%)		
Elementary school or less	8 (10.96)	1 (9.09)
Less than high school	3 (4.11)	0 (0.00)
Completed high school/GED	18 (24.66)	3 (27.27)
Some college (community college/trade school)	23 (31.51)	3 (27.27)
College graduate	19 (26.03)	3 (27.27)
Post-graduate/professional degree	2 (2.74)	1 (9.09)
Parent, Primary Caregiver or Legal Guardian of Child (*n*%)	60 (82.19)	10 (90.91)

^a^ Surveys were only administered to English- or Spanish-speaking customers aged 18 years or more, who purchased a kids’ meal inside the restaurant for a child 12 years old or younger. Child demographics were reported by the participating adult that accompanied the child. If the respondent purchased a kids’ meal for more than one child under 12, they were asked about the oldest child. ^b^ “Standards” refer to any voluntary healthy default beverage standards in place at the QSR at the time of visiting. All sampled QSRs were considered to either be “with standards” or “without standards”. Standards included combinations of flavored and unflavored low-fat, fat-free or reduced-fat milk; 100% juice or “diluted” 42% juice and/or bottled water as the default options with kid’s meals. ^c^ These restaurants had missing data for all menu board items, but were included in the total sample due to having non-missing information about orders. ^d^ Participating adults self-reported their highest level of education and whether they were the parent, primary caregiver and/or legal guardian of the child.

**Table 2:** Kids’ meal beverages displayed on quick-service restaurant (QSR) menu boards, by beverage type (*N* = 149 menu boards total; menu boards of QSRs with standards, *n* = 103; menu boards of QSRs without standards, *n* = 46) ^a^.

Beverages Shown (Listed or Pictured) on Menu Board (*n* = Number of Observations for A Given Beverage Category in QSRs with Standards, Number of Observations for A Given Beverage Category in QSRs without Standards)	Menu Boards of QSRs with Standards ^b^ that Displayed Beverages		Menu Boards of QSRs without Standards ^b^ that Displayed Beverages		*p*-Value ^1^
	*n*	%	*n*	%	
Only unflavored milk or water (as allowed under California legislation) ^c^ (*n* = 97, 46)	15	15.46	0	0.00	NA ^2^
Only milk (unflavored or flavored), water or unsweetened juice (as allowed under voluntary standards) ^d^ (*n* = 86, 46)	56	65.12	2	4.35	**<0.001**
Any milk (*n* = 100, 44)	93	93.00	19	43.18	**0.004**
Unflavored milk (*n* = 100, 44)	92	92.00	19	43.18	**0.005**
Any flavored milk (*n* = 87, 42)	25	28.74	2	4.76	0.407
Any water (*n* = 95, 43)	57	60.00	14	32.56	**0.036**
Any unsweetened juice (*n* = 95, 43)	53	55.79	10	23.26	0.100
Any sugar-sweetened beverage (SSB) ^e^ (*n* = 86, 44)	25	29.07	17	38.64	0.351
Generic “drink”/”kids’ drink” (*n* = 103, 46)	2	1.94	19	41.30	**0.037**
Diet drinks (*n* = 86, 43)	1	1.16	8	18.60	0.144

^a^ A total of 103 menu boards from QSRs with standards and 46 menu boards from QSRs without standards had non-missing data for at least one of the beverage categories presented in this table. The total number of menu boards included in the analysis for each beverage category varies due to missing data for a given beverage category. ^b^ “Standards” refers to any voluntary healthy default beverage standards in place at the QSR at the time of visiting. All sampled QSRs were considered to either be “with standards” or “without standards”. Standards included combinations of flavored and unflavored low-fat, fat-free or reduced-fat milk; 100% juice or “diluted” 42% juice and/or bottled water as the default options with kid’s meals. ^c^ Unflavored milk and water are the only allowable healthy default beverages for kids’ meals as specified by California law SB-1192, implemented after these data were collected. ^d^ Unflavored milk, water or unsweetened juice are common beverages allowed in kids’ meals under restaurants’ voluntary healthy default beverage standards. ^e^ “Sugar-sweetened beverage” includes any beverage with added sweeteners except flavored milk. Beverages included: regular, non-diet soda; energy or sports drinks, non-diet; fruit juice with added sugar; lemonade, fruit punch, aguas frescas, sweet tea or “Fountain drink”. ^1^ *p*-values calculated via multilevel logistic regression, adjusting for clustering by chain and restaurant; bold indicates statistically significant at alpha < 0.05. ^2^ Model unable to converge due to a lack of variation in the data.

**Table 3:** Beverages offered by cashiers during research staff kids’ meal orders at quick-service restaurants (QSRs; *N* = 185 orders).

	Orders at QSRs with Standards ^a^		Orders at QSRs without Standards ^a^		*p*-Value ^1^
	*n*	%	*n*	%	
Beverages Initially Offered by Cashier When Meal Order Placed (*n* = 113 orders at 70 QSRs with standards, 72 orders at 41 QSRs without standards)					
Only unflavored milk and/or water	6	5.31	0	0.00	NA ^2^
Water and/or unflavored milk and other drinks	17	15.04	1	1.39	**0.024**
Beverages other than unflavored milk or water	19	16.81	4	5.56	0.090
Asked what drink (s) the customer wanted with kids’ meal	60	53.10	45	62.50	0.278
Cashier provided a beverage or fountain cup without offering any options (cashier-chosen beverage) ^b^	11	9.73	22	30.56	NA ^2^
**Beverages Offered by Cashier When Asked *What are the options*? or *Are there any other options*? (98 orders at 64 QSRs with standards, 51 orders at 37 QSRs without standards)**					
Unflavored milk	64	65.31	21	41.18	0.161
Any water	35	35.71	16	31.37	0.374
Any juice	64	65.31	24	47.06	0.154
Sugar-sweetened beverages (SSBs) ^c^	69	70.41	45	88.24	0.087
Flavored milk	49	50.00	10	19.61	0.170
Diet drink	38	38.78	30	58.82	**0.042**
None ^d^	5	5.10	0	0.00	NA ^2^

^a^ “Standards” refers to any voluntary healthy default beverage standards in place at the QSR at the time of visiting. All sampled QSRs were considered to either be “with standards” or “without standards”. Standards included combinations of flavored and unflavored low-fat, fat-free or reduced-fat milk; 100% juice or “diluted” 42% juice and/or bottled water as the default options with kids’ meals. ^b^ Response selected by data collectors when cashier did not ask about or offer any beverages, but handed a drink to the data collector. However, what beverage the cashier provided in these instances was not recorded. ^c^ “Sugar-sweetened beverages” include any beverage with added sweeteners, except flavored milk. Beverages included: regular, non-diet soda; energy or sports drinks, non-diet; fruit-flavored drinks (fruit juice with added sugar; lemonade, fruit punch, aguas frescas, sweet tea). ^d^ Cashier said they could not get any other drink with kids’ meal when asked. ^1^ *p*-values calculated via multilevel logistic regression, adjusting for clustering by chain and restaurant; bold indicates statistically significant at alpha < 0.05. ^2^ Model unable to estimate adjusted *p*-value due to a lack of variation in data.

**Table 4:** Characteristics of customer orders placed at quick-service restaurants (QSRs; *N* = 84 customer orders) ^a,b^.

	Total	
	Customer Orders at QSRs with Standards ^c^ (*n* =73)	Customer Orders at QSRs without Standards ^c^ (*n* = 11)
	*n* (%)	*n* (%)
How Order Was Placed		
Inside, with cashier	59 (80.82)	10 (90.91)
Inside, at electronic kiosk	12 (16.44)	0 (0.00)
Using the restaurant app	1 (1.37)	0 (0.00)
Missing	1 (1.37)	1 (9.09)
**Beverage Ordered ^d^**		
Water	0 (0.00)	1 (9.09)
Unflavored milk	5 (6.85)	0 (0.00)
No beverage ordered	0 (0.00)	1 (9.09)
Juice (100% or diluted) ^e^	27 (36.99)	0 (0.00)
Regular, non-diet soda	19 (26.03)	5 (45.45)
Other pre-sweetened drinks ^f^	21 (28.77)	4 (36.36)
Diet drinks ^g^	1 (1.37)	0 (0.00)
Unflavored milk, water or juice ^h^	32 (43.83)	2 (18.18)
Unflavored milk or water ^i^	5 (6.85)	2 (18.18)

^a^ Customers in the sample were interviewed at 26 QSRs with voluntary healthy default beverage standards and 7 QSRs without healthy default beverage standards: 32 chain QSRs and 1 independent QSR. One survey was excluded from analysis. ^b^ Statistical analyses assessing differences between customer orders at QSRs with and without voluntary healthy default beverage standards were not conducted due to the small number of customers sampled from QSRs without any such standards. ^c^ “Standards” refer to any voluntary healthy default beverage standards in place at the QSR at the time of visiting. All sampled QSRs were considered to either be “with standards” or “without standards”. Standards included combinations of flavored and unflavored low-fat, fat-free or reduced-fat milk; 100% juice or “diluted” 42% juice and/or bottled water as the default options with kids’ meals. ^d^ Beverage ordered with kids’ meal purchased for oldest child (if more than one kids’ meal purchased for children 12 or under). ^e^ “Diluted juice” is a 42% juice beverage made by adding water to 100% juice without added sugars. ^f^ “Other pre-sweetened drinks” include sports drinks, Capri Sun, fruit punch, lemonade, aguas frescas, sweet tea and flavored milk. ^g^ “Diet drinks” include diet soda and lite tea. ^h^ Default drinks allowed under many voluntary standards. See footnote (c) above. ^i^ Default drinks for kids’ meals allowed by California state healthy default beverage law (SB-1192) that went into effect after these data were collected.
